# Clinical characteristics, predictive factors, and therapeutic outcomes of mycoplasma pneumoniae pneumonia with pleural effusion in children: a retrospective cohort study

**DOI:** 10.3389/fped.2026.1845802

**Published:** 2026-06-22

**Authors:** Mingfang Liu, Yingjian Cai

**Affiliations:** Department of Pediatrics, The Second Affiliated Hospital of Fujian Medical University, Quanzhou, China

**Keywords:** children, clinical features, mycoplasma pneumoniae, pleural effusion, predictors, treatment outcome

## Abstract

**Objective:**

To analyze the clinical characteristics, predictive factors and curative effects of mycoplasma pneumoniae (MP) infection combined with pleural effusion in children.

**Methods:**

The medical records of 200 children diagnosed with MP pneumonia in our hospital from January 2012 to December 2022 were retrospectively analyzed. According to the presence or absence of pleural effusion, all the children were divided into mycoplasma pneumoniae pneumonia (MPP) with pleural effusion group (100 cases) and MPP without pleural effusion group (100 cases). The differences in clinical manifestations, laboratory indicators, imaging examination, treatment plan and treatment effect between the two groups were compared. Multivariate Logistic regression model was used to analyze the potential predictors of MPP combined with pleural effusion.

**Result:**

Age, length of hospital stay CRP level, white blood cell count, neutrophil count, IgM level, left lung disease, intravenous glucocorticoid use, duration of fever during hospitalization and duration of antibiotic use in MPP group were higher than those in group without pleural effusion (*P* < 0.05). The number of cases, bilateral lung lesions and IgA levels in autumn were significantly lower than those in the group without pleural effusion (*P* < 0.05). Multivariate Logistic regression analysis showed that age, CRP, white blood cell count, autumn infection, IgM and IgA levels were correlated with pleural effusion.

**Conclusions:**

The predictors of MP infection with pleural effusion in children include age, CRP, WBC, autumn infection, IgM and IgA. These factors may contribute to the early identification and treatment of children with MP pneumonia combined with pleural effusion.

## 1 Introduction

Mycoplasma pneumoniae (MP) is a common bacterial respiratory pathogen that causes a high incidence of Community-Acquired Pneumonia (CAP), especially in children (1). MP is a kind of cell wall free, moderate sized microorganism, whose special biological characteristics enable it to survive and multiply in the host cell, and may cause a range of respiratory diseases, including mild pharyngitis to more serious respiratory infections such as pneumonia (2). The clinical manifestations of mycoplasma pneumoniae infection in children vary from mild dry cough and low fever to high fever, cough, headache and general malaise. Serious complications, such as pleural effusion, may indicate worsening of the patient's condition (3, 4).

In recent years, cases of MP pneumonia in children have been reported more and more frequently, and some of them are severe due to pleural effusion, which poses a significant burden on the health and quality of life of children (5). Pleural effusion is a buildup of fluid in the pleural cavity caused by a variety of causes, which may be caused by heart, kidney, or liver disease, as well as infections, tumors, or other diseases (6). In the context of MP infection, cases with pleural effusion may require close medical observation and special therapeutic interventions, including medication or pleural effusion aspiration, to prevent disease progression and reduce the risk of complications (7, 8).

Although many studies have focused on the occurrence and complications of MP pneumonia in adults, there are relatively few systematic studies on MP infection and pleural effusion in children (9). In addition, MP resistance to macrolide antibiotics has been reported globally, especially in Asia, where the proportion of resistance cases is increasing. A recent study from Beijing, China, involving 8,453 children with respiratory tract infections between 2018 and 2024, reported that the proportion of macrolide-resistant MP reached as high as 96.9%, and remained consistently above 95% after 2022 (10). This suggests that traditional antibiotic treatment protocols may no longer be applicable and new treatments need to be explored. Therefore, it is necessary to recognize this situation and adopt appropriate strategies in the treatment of children with MP infection.

Based on medical record data, this study analyzed the clinical features, predictors and treatment outcomes of mycoplasma pneumoniae infection combined with pleural effusion in children through retrospective study design. By comparing children with and without pleural effusion, the aim is to identify biomarkers and risk factors that may predict the occurrence of complications in order to identify high-risk patients in a timely manner at an early stage and provide them with appropriate therapeutic interventions.

## 2 Data and methods

### 2.1 General information

This is a retrospective study of children diagnosed with mycoplasma pneumoniae pneumonia in the pediatric ward of our hospital between January 2012 and December 2022. During the study period, a total of 200 children were included and divided into 2 groups according to the presence or absence of pleural effusion, of which 100 were MPP with pleural effusion group and 100 were MPP without pleural effusion group. In MPP without pleural effusion group, the ratio of male to female was 53:47, and the mean age was 5. 10 ± 2.59 years. The male to female ratio in MPP patients with pleural effusion was 63:37, and the mean age was 6.35 ± 2.51 years.

Inclusion criteria: children between 1 and 14 years of age; Diagnosis of mycoplasma pneumonia: symptoms, signs, laboratory tests and x-ray chest films meet the diagnostic criteria for mycoplasma pneumoniae pneumonia; Mycoplasma pneumoniae antibody titer increased or PCR positive; Patients' electronic medical records are complete according to standards and provide detailed clinical and laboratory data; Parents or guardians sign informed consent forms.

Exclusion criteria: evidence of bacterial or viral pneumonia other than mycoplasma pneumoniae; Complicated with severe extrapulmonary infection, such as sepsis, meningitis, pericarditis, etc. Have an underlying medical condition, such as immune deficiency, chronic lung disease, heart disease or other chronic disease; use of antibiotics (for any reason, including respiratory infection or other conditions) within 2 weeks prior to admission; Adequate data on the children were not available at the start of the study, or the children were ineligible for follow-up for other reasons.

### 2.2 Methods

By analyzing the collected data of children, this retrospective study compared the clinical manifestations, laboratory results, and therapeutic effects of mycoplasma pneumoniae infection combined with pleural effusion and mycoplasma pneumoniae infection alone, analyzed potential predictors, and evaluated the effects of different treatments.

#### 2.2.1 Collect information of children

This study collected data of children diagnosed with mycoplasma pneumoniae pneumonia in the pediatric ward of our hospital from January 2012 to December 2022, including age, sex, time of first onset of symptoms, specific symptoms, drug use, duration of fever during hospitalization, and duration of antibiotic use.

#### 2.2.2 Physical examination

Detailed physical examination was performed on each child after admission, including observation of mental state, assessment of respiratory function, observation of chest wall activity, tapping and auscultation of lungs, etc.

#### 2.2.3 Blood, imaging diagnosis and sputum culture

5 mL of fasting venous blood was collected from all children, and the blood routine was detected by automatic hematology analyzer (Sysmex XN-1000, Sysmex Corporation, Kobe, Japan). CRP levels were measured by chemiluminescent immunoassay (Siemens ADVIA Centaur, Siemens Healthcare Diagnostics, Tarrytown, NY, USA). Immunoglobulin was detected by the visible immunoturbidimetric method with spectrophotometer (Beckman Coulter IMMAGE 800, Beckman Coulter, Brea, CA, USA). Imaging tests were performed by chest x-rays and CT scans, which were read and agreed upon by two experienced physicians. Disposable sterile sputum aspirators were used to collect sputum from all children on the day of admission, and the sputum was inoculated in AGAR medium or chocolate medium within 30 min, and bacterial culture was conducted in 5% CO2 incubator. After 24 h, according to the bacterial growth, automatic microbial analyzer was used to identify the sputum.

#### 2.2.4 Disease course, stage and treatment plan

Treatment options include antimicrobial therapy, supportive therapy (e.g., oxygen therapy, nutritional support, etc.) and, if necessary, pleural effusion pumping or other interventions. Methods of treatment, changes during treatment, length of hospital stayand follow-up were collected for each child.

### 2.3 Observation indicators

The main outcome measures of this study included clinical manifestations, laboratory findings, imaging features and treatment response. All children underwent blood and imaging tests, including blood routine, C-reactive protein (CRP), and immunoglobulin (IgG, IgM, and IgA) tests. Imaging tests include chest radiographs and chest CT scans to determine the location of lung lesions and the presence of pleural effusion. In terms of treatment response, the specific method of treatment received by each child, the number of days in hospital and the changes in the condition during follow-up were recorded.

### 2.4 Statistical analysis

Descriptive statistical analysis was performed on all data, and mean and standard deviation were calculated for quantitative data. Comparisons between the two sets of data were made using either the independent sample *t*-test or the Mann–Whitney *U*-test, selected according to the distribution of the data. Categorical variables are analyzed by Chi-square test or Fisher precision test. For possible risk factors, Logistic regression models were used to assess their association with pleural effusion combination, and corresponding odds ratios (ORs) and 95% confidence intervals (CIs) were calculated. All statistical tests were conducted by bilateral tests, and *P* < 0.05 was considered statistically significant. All data were analyzed using the statistical package SPSS (Version 25.0, SPSS Inc., Chicago, IL, USA).

## 3 Results

### 3.1 Baseline characteristics and clinical manifestations

There was no significant difference in the ratio of male to female in MPP with pleural effusion group compared with MPP without pleural effusion group (*P* > 0.05). However, the age of the patients with pleural effusion was longer than that without pleural effusion. In addition, the mean hospital stay of children with pleural effusion was longer than that of those without pleural effusion. Laboratory examination showed that the white blood cell count, CRP level and neutrophil percentage (*N*%) in the group with pleural effusion were significantly higher than those in the group without pleural effusion (*P* < 0.05). The number of children with pleural effusion in autumn was significantly lower than that without pleural effusion (*P* < 0.001). There was no significant difference in condensing between the two groups (*P* > 0.05), as shown in Table 1.

**Table 1:** Comparison of baseline features and clinical findings.

Variable	MPP without pleural effusion group (*n* = 100)	MPP with pleural effusion group (*n* = 100)	*t*/*χ*^2^	*P*
Gender (male/female) [*n* (%)]	53 (53%)/47 (47%)	63 (63%)/37 (37%)	2.053	0.152
Age (years)	5.10 ± 2.59	6.35 ± 2.51	−3.466	0.001
Length of hospital stay (days)	5.98 ± 2.14	7.26 ± 3.04	−3.443	0.001
White blood cells (×10^9^/L)	8.59 ± 1.36	9.37 ± 3.58	−2.037	0.043
CRP (mg/L)	9.90 ± 7.01	30.22 ± 24.01	−8.124	<0.001
N% Seasonal distribution	56.99 ± 11.22	66.01 ± 11.90	−5.515	<0.001
Spring [*n* (%)]	14 (14%)	21 (21%)	2.512	0.113
Summer [*n* (%)]	16 (16%)	25 (25%)	3.182	0.075
Autumn [*n* (%)]	42 (42%)	17 (17%)	8.947	<0.001
Winter [*n* (%)]	28 (28%)	37 (37%)	1.854	0.174
Condensing (positive/negative) [*n* (%)]	34 (34%)/66 (66%)	36 (36%)/64 (64%)	0.022	0.882

### 3.2 Imaging and laboratory results

Imaging results showed that lung lesions were more likely to occur on the left side in the group with pleural effusion, while bilateral lesions were more common in the group without pleural effusion (*P* < 0.001). There was no significant difference in bronchoscopy and sputum culture between the two groups (*P* > 0.05). Laboratory results showed that the levels of immunoglobulin M (IgM) and immunoglobulin A (IgA) were significantly different between the two groups. IgM was higher in the group with pleural effusion, IgA was higher in the group without pleural effusion, and immunoglobulin G was not significantly different (*P* > 0.05), as shown in Table 2.

**Table 2:** Imaging and laboratory results.

Parameter	MPP without pleural effusion group (*n* = 100)	MPP with pleural effusion group (*n* = 100)	*t*/χ^2^	*P*
Distribution of pulmonary lesions [*n* (%)]—left side	19 (19%)	40 (40%)	6.237	0.012
Distribution of pulmonary lesions [*n* (%)]—right side	35 (35%)	48 (48%)	2.840	0.092
Distribution of pulmonary lesions [*n* (%)]—both sides	46 (46%)	12 (12%)	17.005	<0.001
Bronchoscopy—Congestion [*n* (%)]	32 (89%)	36 (73%)	2.751	0.097
Bronchoscopy mucus plug [*n* (%)]	4 (11%)	13 (27%)	3.448	0.063
Sputum culture Streptococcus [*n* (%)]	5 (71%)	4 (80%)	3.141	0.076
Phlegm culture—Catalamolla [*n* (%)]	2 (29%)	1 (20%)	1.258	0.262
Ig G (mg/dL)	958.46 ± 2 33.52	986.060 ± 313.741	−0.706	0.481
Ig M (mg/dL)	130.48 ± 7 7.65	167.390 ± 85.684	−3.192	0.002
IgA (mg/dL)	195.51 ± 7 9.93	164.497 ± 87.218	2.621	0.009

### 3.3 Drug treatment plan

In terms of the use of medication regimens, the MPP with pleural effusion group used intravenous glucocorticoids more frequently than the MPP without pleural effusion group (*P* < 0.05). Other medication regimens, including macrolide antibiotics alone, combined therapy with macrolides and β-lactam drugs, and intravenous propyl spheres showed no significant difference between the two groups, as shown in Table 3.

**Table 3:** Post-admission medication regimen [*n* (%)].

Medication regimen	MPP without pleural effusion group (n = 100)	MPP with pleural effusion group (*n* = 100)	χ^2^	*P*
Macrolides were used alone	71 (71%)	59 (59%)	2.659	0.103
Combined with treatment macrolides and beta-lactam drugs	27 (27%)	35 (35%)	1.145	0.285
Intravenous glucocorticoids	12 (12%)	31 (31%)	9.599	0.002
intravenous immunoglobulin	0 (0%)	3 (3%)	-	-

### 3.4 Treatment response and clinical improvement

In terms of treatment response, the duration of fever and antibiotic use during hospitalization in the patients with pleural effusion group were significantly longer than those without pleural effusion group (*P* < 0.001), as shown in Table 4.

**Table 4:** Comparison of treatment response and clinical improvement.

Outcome measure	MPP without pleural effusion group (*n* = 100)	MPP with pleural effusion group (*n* = 100)	*t*	*P*
Duration of fever during hospitalization (days)	2.50 ± 1.20	3.50 ± 1.40	5.074	<0.001
Duration of antibiotic use during hospitalization (days)	7.00 ± 2.00	9.00 ± 2.50	6.400	<0.001

### 3.5 Multivariate logistic regression analysis

Table 5 presents the predictors of mycoplasma pneumoniae pneumonia combined with pleural effusion in children through multivariate Logistic regression analysis.

**Table 5:** Multivariate logistic regression analysis.

Variable	*Β* value	SE	OR	95% CI	*P*
Age	0.113	0.031	1.122	1.051–1.203	0.001
CRP	0.221	0.054	1.255	1.132–1.391	<0.001
White blood cell count	0.091	0.046	1.092	1.011–1.184	0.028
Season (Autumn)	–0.391	0.162	0.686	0.491–0.942	0.021
IgM	0.318	0.281	1.795	1.777–4.867	0.00
IgA	0.267	0.288	0.281	0.131–0.602	0.001

**Figure 1:**
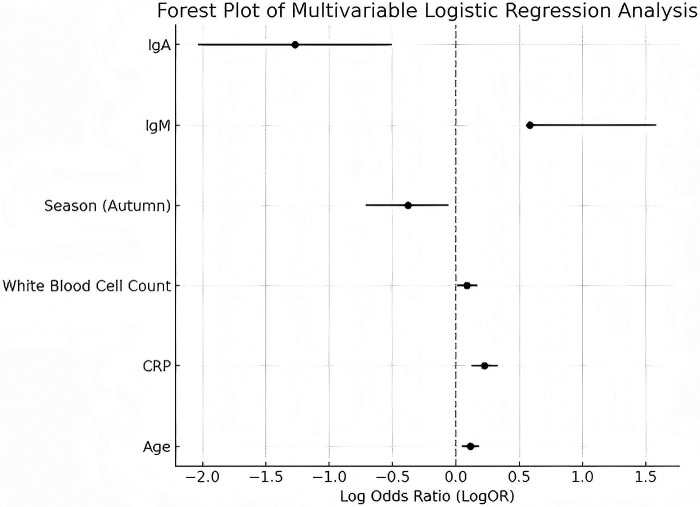
Multivariate logistic regression analysis.

The analysis revealed that the risk of pleural effusion increased by 12.2% for each additional year of age (OR = 1.122, 95% CI: 1.051–1.203, *P* = 0.001). For every 10 mg/L increase in CRP, the risk increased by 25.5% (OR = 1.255, 95% CI: 1.132–1.391, *P* < 0.001). For every 1 × 10^9^/L increase in white blood cell count, the risk increased by 9.2% (OR = 1.092, 95% CI: 1.011–1.184, *P* = 0.028). The risk of pleural effusion was lower in autumn than in other seasons (OR = 0.686, 95%CI: 0.491–0.942, *P* = 0.021). In addition, an increase in immunoglobulin M (IgM) significantly increased the risk of co-pleural effusion (OR = 1.795, 95% CI: 1.777–4.867, *P* = 0.001), while an increase in immunoglobulin A (IgA) significantly decreased the risk (OR = 0.281, 95% CI: 0.131–0.602, *P* = 0.001), as shown in Figure 1.

## 4 Discussion

Mycoplasma pneumoniae (MP) is the smallest pathogenic microorganism, between bacteria and viruses, able to survive autonomously in a cell-free culture medium, lacking the structure of cell walls. MP is one of the common causes of community acquired pneumonia (CAP) in children in China (11). It is noteworthy that the number of cases of severe mycoplasma pneumoniae pneumonia (SMPP) has been increasing year by year (12). Children with SMPP are at high risk of developing serious diseases such as atelectasis, pleural effusion, lung abscess, and pulmonary fibrosis, which can be life-threatening in extreme cases. In Asia, especially in China, the incidence rate of MP pneumonia resistant to macrolide is as high as 80%–90%, and the proportion of MP pneumonia resistant to macrolide in China is as high as 95%, which leads to increasingly prominent problems such as reduced efficacy of macrolide antibiotics, difficult to control symptoms and increasing complications (13, 14).

In view of this, it is critical to explore the risk factors for pleural effusion in patients with mycoplasma pneumoniae pneumonia and analyze whether combination therapy with macrolides and beta-lactam drugs can reduce the occurrence of pleural effusion. At present, there is no clear standard on the risk factors of severe mycoplasma pneumoniae pneumonia associated with pleural effusion at home and abroad. A study of 347 children diagnosed with mycoplasma pneumonia found that childhood age, history of recurrent respiratory infection, hypersensitive C-reactive protein level, and sedimentation rate were risk factors for severe mycoplasma pneumonia (15). In addition, 23s rRNA gene detection conducted on 96 MPP children showed that the proportion of drug resistance was as high as 84%, and the drug-resistant strains were mainly P1-I and M4-5-7-2. This research result may provide a new drug target for the treatment of mycoplasma infection in the future (16). In another retrospective analysis of pediatric cases of mycoplasma pneumoniae pneumonia, P1-restricted fragment length polymorphism and multi-site variable number tandem repeat analysis were used to identify MP-positive samples, identify Mp strains associated with increased risk of SMPP and pleural effusion, and simultaneously detect macrolide resistance mutations. This provides new laboratory parameters for early identification of SMPP (17).

By comparing the clinical features, laboratory and imaging findings and treatment responses of MPP patients without pleural effusion and MPP patients with pleural effusion, this study aims to explore the predictive factors and treatment outcomes of MPP infection with pleural effusion.

Analysis of clinical features and predictors showed that CRP level and neutrophil percentage in MPP with pleural effusion group were significantly higher than those in MPP without pleural effusion group. Consistent with previous studies, Ling, et al. (6) found that patients with mycoplasma infection combined with pleural effusion had higher CRP levels. Multivariate Logistic regression analysis revealed multiple independent predictors of combined pleural effusion, including age, CRP level, white blood cell count, and immunoglobulin level.

The analysis of seasonal distribution showed that more children with pleural effusion were treated in spring and summer, which was consistent with the high incidence of mycoplasma infection in previous years. In addition, the risk of pleural effusion caused by infection in autumn is lower, which may be related to seasonal environmental factors or pathogen virulence, but more research is needed to explore the specific mechanisms. This study also found that IgM and IgA levels in children were associated with the risk of pleural effusion. Higher IgM levels were associated with an increased risk of complicating pleural effusion, while increased IgA was associated with a reduced risk, suggesting that the host immune response to MPP plays a key role in the disease process. These results are consistent with the study by Lee, et al. (18), who reported the potential value of IgM and IgA in MPP diagnosis and in determining disease severity. In clinical practice, IgM and IgA levels are not routinely measured in all children with pneumonia; however, our findings suggest that these immunoglobulins may serve as useful adjunctive markers for risk assessment and could be considered more often in suspected severe cases.

This study has some limitations. Due to the nature of retrospective studies, there is potential for selection bias. Second, the relatively small sample size may have limited the strength of the statistical analysis. In addition, studies focused on a single center and may not be able to fully generalize all populations. Furthermore, this study did not differentiate between simple parapneumonic effusion and empyema, a rarer complication of Mycoplasma pneumoniae infection. Future studies with detailed pleural fluid analysis are warranted to explore whether empyema has distinct predictive factors and outcomes. Future studies may consider including a wider geographical sample and adopt a prospective design to increase the representativeness of the data and the generalization of the conclusions.

In summary, there were significant differences in clinical features and laboratory parameters between MPP patients with pleural effusion and those without, and variables such as age, CRP, white blood cell count, autumn infection, IgM, and IgA levels could be used as predictors of pleural effusion. Fully recognizing the clinical value of these predictors will help facilitate the development and improvement of treatment strategies for this specific patient population.

## Data Availability

The original contributions presented in the study are included in the article/Supplementary Material, further inquiries can be directed to the corresponding author.
